# Celecoxib alleviates nonalcoholic fatty liver disease by restoring autophagic flux

**DOI:** 10.1038/s41598-018-22339-0

**Published:** 2018-03-07

**Authors:** Cong Liu, Lian Liu, Hai-Dan Zhu, Jia-Qi Sheng, Xiao-Li Wu, Xing-Xing He, De-An Tian, Jia-Zhi Liao, Pei-Yuan Li

**Affiliations:** Division of Gastroenterology, Tongji Hospital, Tongji Medical College, Huazhong University of Science and Technology, Wuhan, 430030 China

## Abstract

Nonalcoholic fatty liver disease (NAFLD) is a kind of liver lipid synthesis and degradation imbalance related with metabolic syndrome. Celecoxib shows the function of ameliorating NAFLD, but the underlying mechanisms remain unknown. Here, we discuss the possible mechanisms of celecoxib alleviating NAFLD by restoring autophagic flux. Lipids were accumulated in L02 cells treated with palmitate as well as SD rats fed with high-fat diet. Western blot showed that LC3 II/I was higher and p62 was lower on the early stage of steatosis while on the late stage both of them were higher, indicating that autophagic flux was activated on the early stage of steatosis, but blocked on the late stage. Rapamycin alleviated steatosis with activating autophagic flux while chloroquine aggravated steatosis with inhibiting autophagic flux. COX-2 siRNA and celecoxib were used to inhibit COX-2. Western blot and RFP-GFP-LC3 double fluorescence system indicated that celecoxib could ameliorate steatosis and restore autophagic flux in L02 cells treated with palmitate as well as SD rats fed with high-fat diet. In conclusion, celecoxib partially restores autophagic flux via downregulation of COX-2 and alleviates steatosis *in vitro* and *in vivo*.

## Introduction

Due to the modern sedentary and food-abundant lifestyle, nonalcoholic fatty liver disease (NAFLD) has become one of the most common liver diseases around world^1^. Prevalence of NAFLD ranges from 5% to 40% in Asia, among which China has become one of the highest prevalence regions^2^. It is widely acknowledged that the spectrum of NAFLD extends from nonalcoholic fatty liver (NAFL) to nonalcoholic steatohepatitis (NASH) and liver cirrhosis^3,4^. Furthermore, NAFLD can progress to liver cancer without fibrosis. Apart from lifestyle changes, effective treatment of NAFLD is still elusive. Recent studies suggest that celecoxib plays an important role in the treatment of NAFLD^5^.

Three types of cyclooxygenase exist, namely COX-1, COX-2, and COX-3^6,7^, among which COX-2 is widely upregulated in NAFLD animal models and steatosis hepatocytes^8,9^. Recent studies reported that the upregulated expression of COX-2 has intertwined relationship with metabolic disorders including obesity, diabetes mellitus, and NAFLD. Furthermore, COX-2 activity not only has influence on insulin sensitivity^10^, but also acts as pro-inflammatory mediator during the progression of NAFLD^11^. Recently, it has gained increasing attention on the mechanisms by which celecoxib protects against the development of NAFLD. Many studies suggested that celecoxib could attenuate liver steatosis and inflammation in NAFLD^5^. Celecoxib, a selective COX-2 inhibitor, may protect cells from palmitate (PA) -induced insulin signaling Akt phosphorylation^10,12,13^. Celecoxib may also alleviate liver inflammation through regulation of NF-kB pathway as well as steatosis via suppression of non-canonical Wnt signaling pathway^14^. In addition, celecoxib may inhibit hepatocytes apoptosis through Akt/p53 signal pathway in NAFLD^12^.

Autophagy, a self-digestion process, plays an important role in the development of NAFLD^15–17^. In fact, apart from the tricarboxylic acid cycle in mitochondria, autophagy is also involved in lipid metabolism namely the lipophagy^18,19^. Lipophagy is a complex multi-step process which refers to the degradation of extra lipid by autolysosomes in cells^16^. Thus, autophagy is quite essential for the balance of lipid metabolism. Recent studies suggested that impaired autophagic flux is associated with the progress of NAFLD^20,21^. Activating autophagy with rapamycin (mTOR inhibitor) will attenuate steatosis, fibrosis, inflammation, ER stress, while treating with chloroquine will make steatosis and liver injury worsen^22^. Although many studies proposed different mechanisms of celecoxib on alleviating NAFLD, none of them have explored whether autophagy participates in this effect. Further studies are needed to clarify the underlying mechanisms of celecoxib on alleviating NAFLD which may provide us a better understanding of the pathogenesis and treatment of NAFLD.

In this study, we try to figure out whether celecoxib attenuates hepatic steatosis by regulating autophagic flux both *in vivo* and *in vitro*.

## Results

### PA induced lipid accumulation in hepatocytes

**Figure 1:**
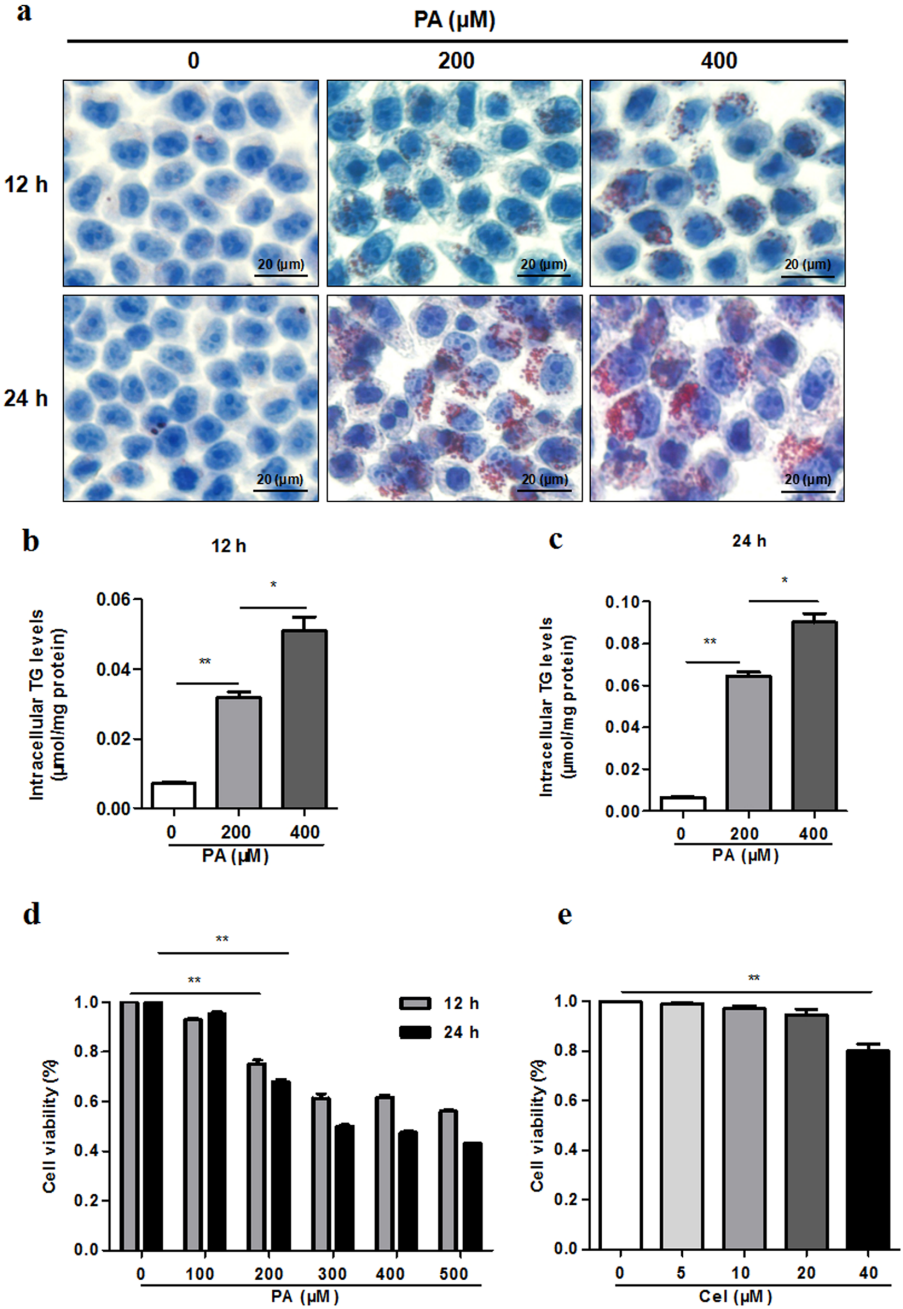
PA induces lipid accumulation in hepatocytes. (**a–c**) L02 hepatic cells exposed to different concentrations (200 μM, 400 μM) of PA for different time (12 h, 24 h). Lipid droplets accumulation and TG levels were determined by Oil Red O staining (400X) and TG assay respectively (*P < 0.05, **P < 0.01). (**d**) L02 cells exposed to different concentrations (100–500 μM) of PA for different time (12 h, 24 h). CCK-8 assay was used to detect cell viability (*P < 0.05, **P < 0.01). (**e**) L02 cells were treated with celecoxib at different concentrations (5–40 μM) for 24 h. CCK-8 assay was used to detect cell viability (*P < 0.05, **P < 0.01).

Oil Red O staining and TG kit were used to examine the levels of lipid accumulation in L02 cells after treating with PA. When compared to the control, intracellular lipid droplets were elevated in L02 cells in a time and dose-dependent manner (Fig. 1a). After treating with different concentrations and time of PA, the TG levels were also remarkably increased in L02 cells (Fig. 1b,c). We examined the effects of PA or celecoxib on cell viability, indicating that PA significantly reduced cell viability from 200 μM (Fig. 1d), while celecoxib had little effect on cell viability within 0–20 μM (Fig. 1e).

### Effects of PA on autophagic flux and COX-2

**Figure 2:**
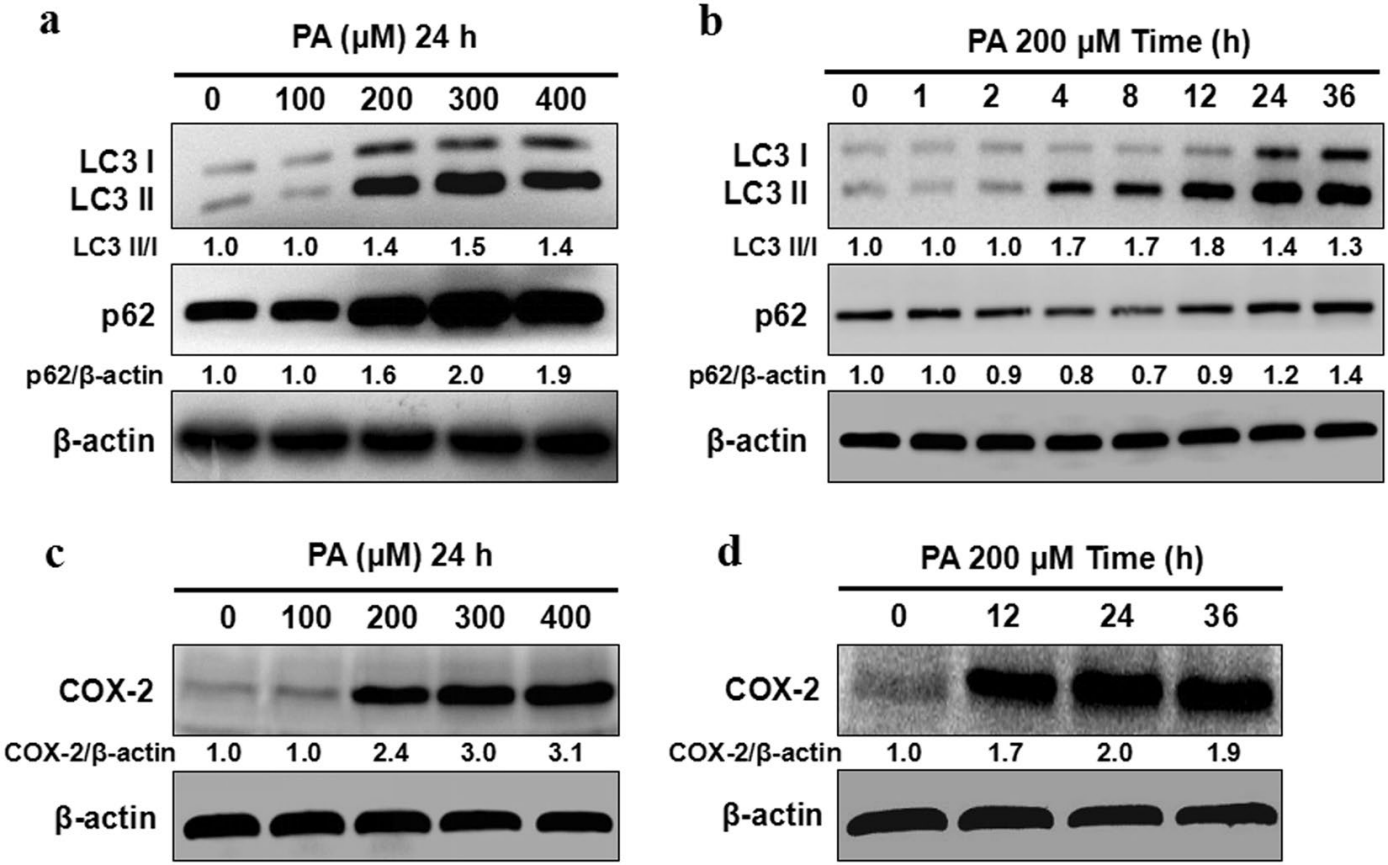
Effects of PA on autophagic flux and COX-2. (**a**) L02 cells exposed to different concentrations (100–400 μM) of PA for 24 h. PA induced upregulation of LC3 II/I and p62 protein levels as indicated by western blot. (**b**) L02 cells were treated with PA (200 μM) for different time (0–36 h). PA induced upregulation of LC3 II/I and downregulation of p62 protein levels at 4 h, 8 h while upregulation of LC3 II/I and p62 protein levels at 24 h, 36 h indicating that autophagic flux was activated on the early stage but blocked at the late hour. (**c,d**) L02 cells were treated with PA of different concentrations (100–400 μM) for different time (12–36 h). PA induced upregulation of COX-2 protein levels. The value under the band is the ratio of the blot and normalized to control.

To investigate the effects of PA on autophagic flux in L02 cells, LC3 II/I and p62 expression, which had been widely acknowledged as hallmarks of autophagic flux, were detected by western blot. Protein expression of LC3 II/I and p62 was increased in L02 cells treated with PA within 200–400 μM for 24 h (Fig. 2a and Supplementary Fig. S1). As shown in Fig. 2b and Supplementary Fig. S2, a remarkable increase of LC3 II/I and reduction of p62 were found in L02 cells treated with 200 μM PA at 4 h and 8 h. While at 24 h and 36 h, both of LC3 II/I and p62 were increased, which indicated that autophagic flux was activated on the early stage of steatosis but blocked at the late hour. The COX-2 expression was also noticeablely increased in L02 cells treated with PA (Fig. 2c,d and Supplementary Fig. S3).

### Autophagy played an important role in steatosis

**Figure 3:**
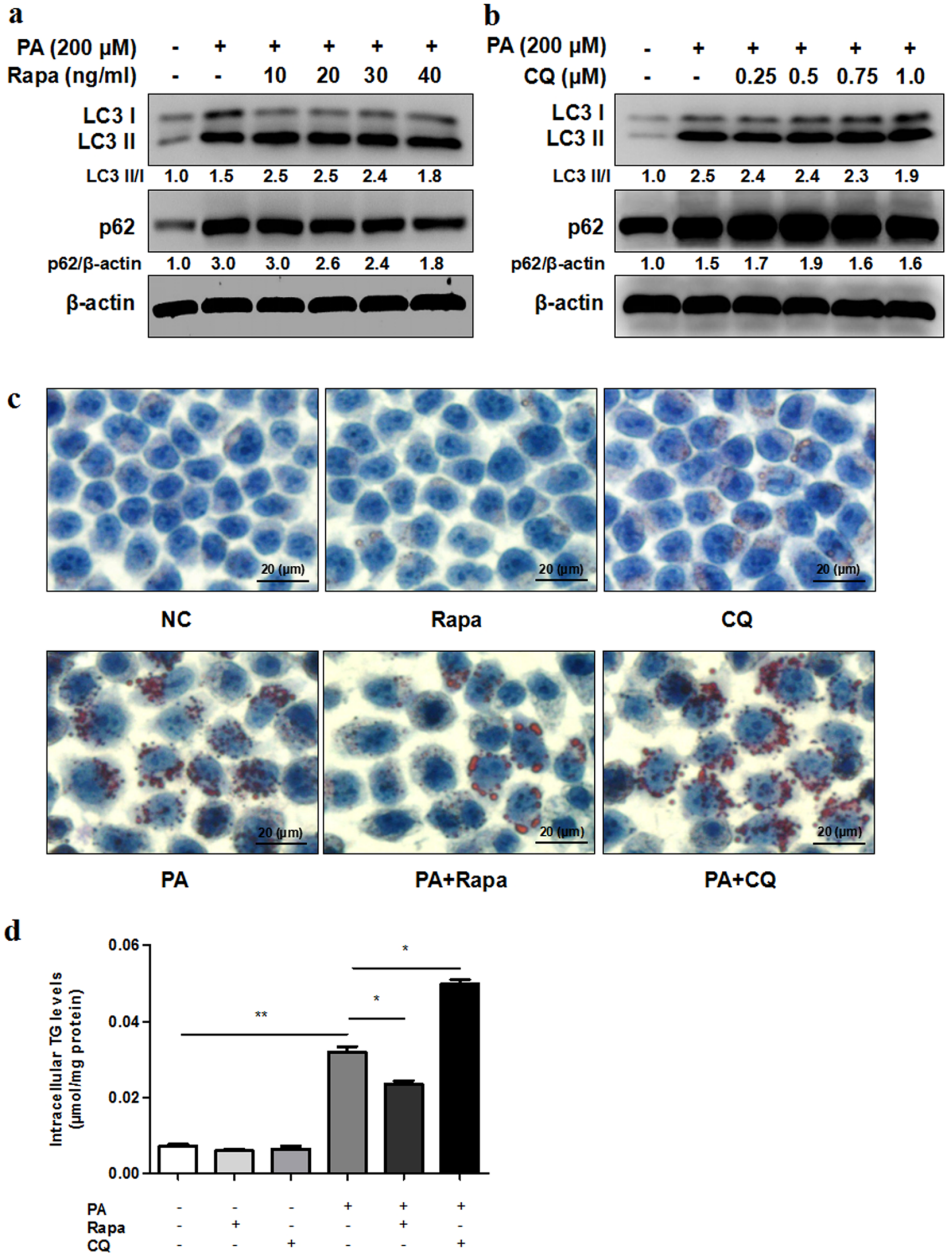
Autophagy plays an important role in steatosis. (**a,b**) L02 cells exposed to PA (200 μM) with different concentrations of rapamycin (Rapa) or chloroquine (CQ) for 24 h. PA induced higher protein expression of LC3 II/I and p62 compared with control as indicated by western blot. Rapamycin combined with PA treatment induced higher LC3 II/I protein levels and lower p62 protein levels compared with PA treatment. Chloroquine combined with PA treatment induced higher p62 protein levels compared with PA treatment. The value under the band is the ratio of the blot and normalized to control. (**c,d**) L02 cells exposed to PA (200 μM) with rapamycin (20 ng/ml) or chloroquine (0.5 μM) for 24 h. Intracellular lipid droplets accumulation and TG levels were determined by Oil Red O staining (400X) or TG assay respectively (*P < 0.05, **P < 0.01). L02 steatosis was decreased with rapamycin treatment while increased with chloroquine treatment.

We used rapamycin (autophagy activator) and chloroquine (autophagy inhibitor) to explore the effect of autophagy on lipid accumulation in steatosis L02 cells induced by PA. Rapamycin, a mammalian target of mTOR inhibitor, has been widely used as autophagy inducer. Rapamycin treatment enhanced the expression of LC3 II/I and reduced the expression of p62 in PA treated L02 cells (Fig. 3a and Supplementary Fig. S4). Meanwhile, lipid droplets accumulated in L02 cells treated with PA as well as TG content displayed a distinct lower level in combination of rapamycin and PA treatment groups than those in PA treatment groups (Fig. 3c,d). Chloroquine is widely acknowledged as a classical autophagy inhibitor with the effect to disrupt the fusion of autophagosome with lysosome and raise of lysosome pH. We found that chloroquine treatment resulted in increase of p62 in PA treated L02 cells (Fig. 3b and Supplementary Fig. S5). Furthermore, lipid droplets accumulation and TG content were remarkably increased in combination of chloroquine and PA treatment groups than those in PA treatment groups (Fig. 3c,d).

### Downregulation of COX-2 induced autophagic flux *in vitro*

**Figure 4:**
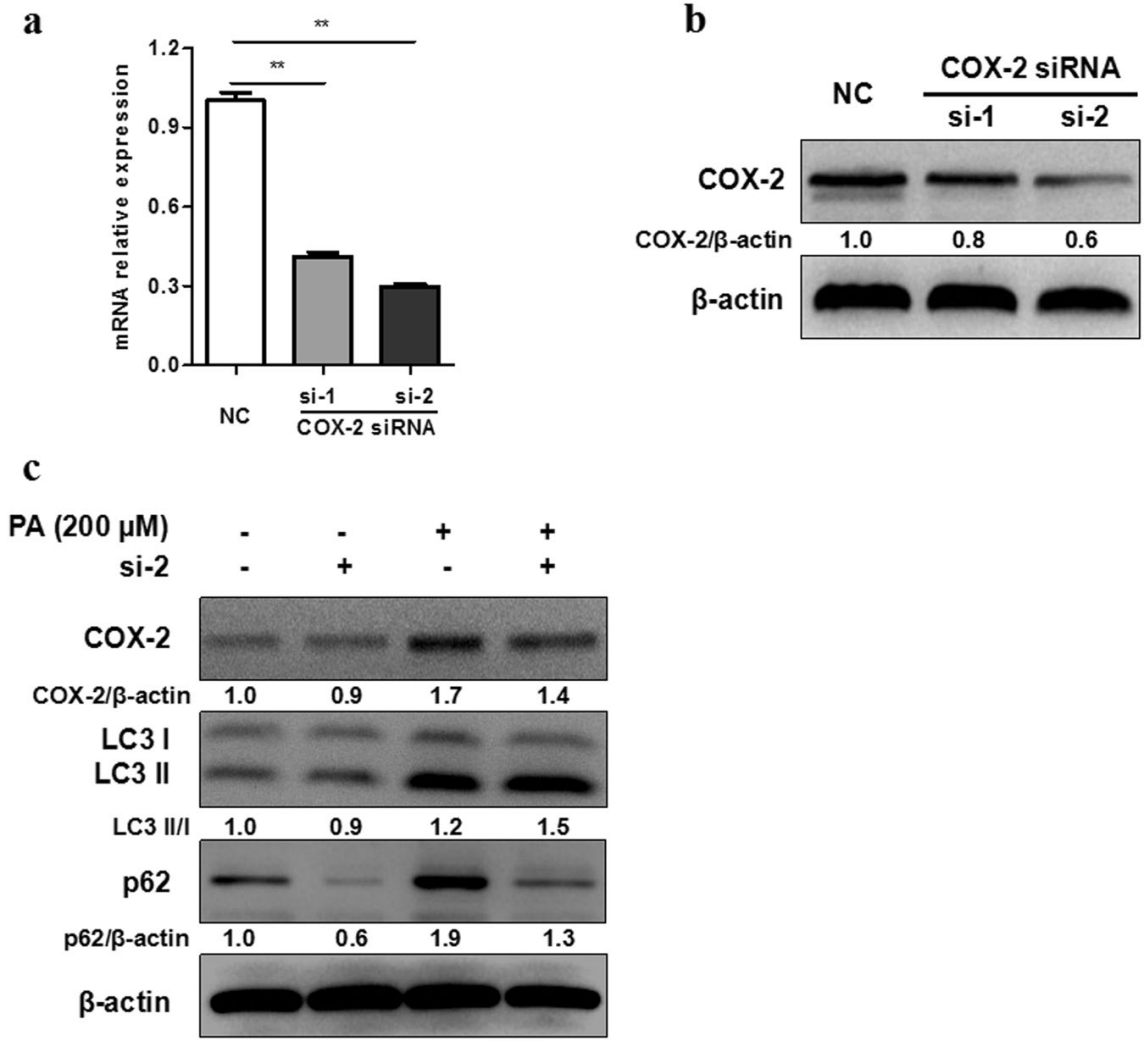
Downregulation of COX-2 induces autophagic flux *in vitro*. (**a,b**) COX-2 was knocked down with COX-2 siRNA transfection (si-1 or si-2) in L02 cells, then RT-PCR and western blot were performed for confirmation. (**c**) After treated with PA (200 μM) for 24 h, western blot of COX-2 siRNA transfected cells showed higher protein expression of LC3 II/I and lower protein expression of p62 than negative control (NC). The value under the band is the ratio of the blot and normalized to control.

To investigate whether an increased expression of COX-2 in steatosis L02 cells induced by PA had an effect on autophagy, we performed siRNA to silence COX-2 expression. As displayed, the mRNA and protein expression of COX-2 were lower in COX-2 siRNA transfected cells than control cells (Fig. 4a,b). When COX-2 was silenced, LC3 II/I was increased and p62 was decreased in L02 cells treated with PA (Fig. 4c and Supplementary Fig. S7).

### Celecoxib alleviated steatosis partially by restoring autophagic flux *in vitro*

**Figure 5:**
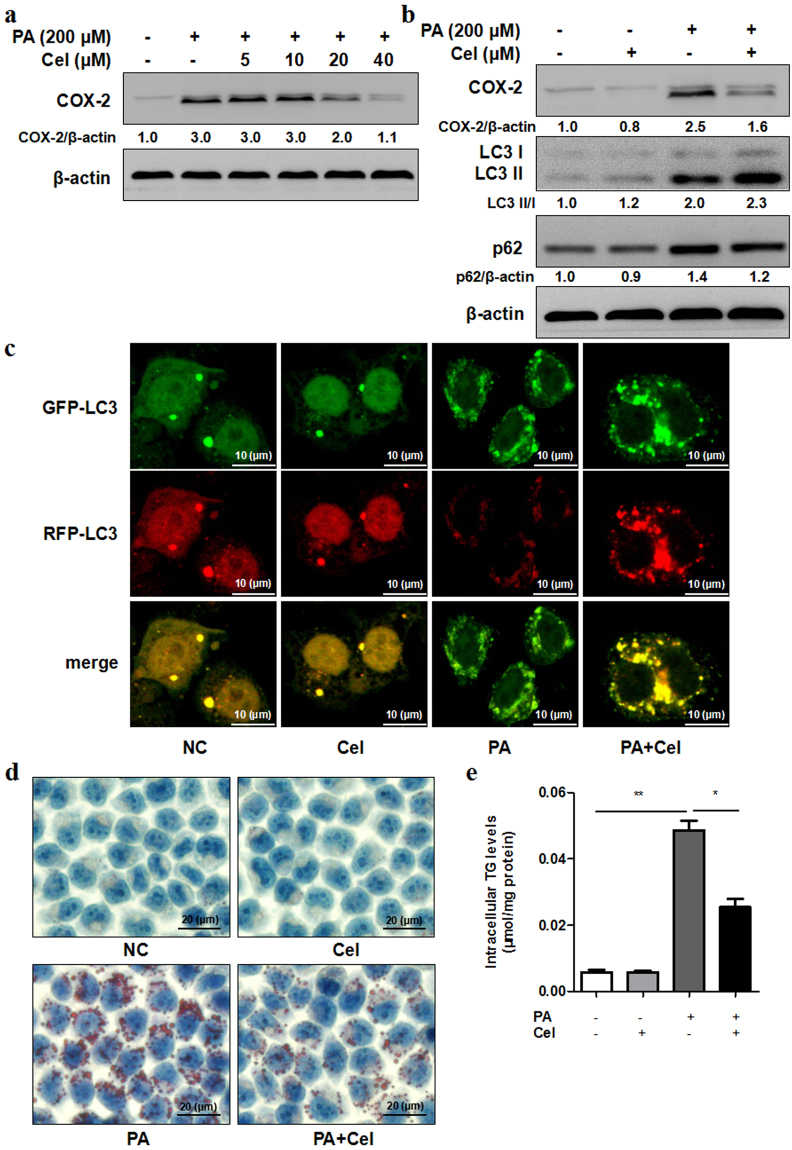
Celecoxib alleviates steatosis by restoring autophagic flux *in vitro*. (**a**) L02 cells exposed to PA (200 μM) with different concentrations of celecoxib (Cel, 5–40 μM) for 24 h. Celecoxib decreased protein expression of COX-2 compared with control as indicated by western blot. (**b**) L02 cells were treated with PA (200 μM) and celecoxib (20 μM) for 24 h. Celecoxib combined with PA treatment induced higher protein expression of LC3 II/I and lower protein expression of p62 compared to PA treatment. The value under the band is the ratio of the blot and normalized to control. (**c**) RFP-GFP-LC3 double fluorescence lentivirus tranfected L02 cells were treated with PA (200 μM) and celecoxib (20 μM) for 24 h. Representative confocal images of GFP-LC3 puncta (green, autophagosomes) and RFP-LC3 puncta (red, autolysosomes), as well as overlay images were shown (1000X). (**d,e**) L02 cells exposed to PA (200 μM) with celecoxib (20 μM) for 24 h. Intracellular lipid droplets accumulation and TG levels were determined by Oil Red O staining (400X) or TG assay respectively (*P < 0.05, **P < 0.01).

Recent studies have shown that celecoxib may protect against the development of NASH by attenuating liver steatosis and inflammation. To determine whether celecoxib alleviates steatosis by regulating autophagic flux *in vitro*, L02 cells were treated with 200 μM PA for 24 h combined with celecoxib. As shown in Fig. 5a and b, protein levels of COX-2 and p62 were remarkably reduced and LC3 II/I was increased. Autophagic flux was further evaluated in RFP-GFP-LC3 lentivirus transfected L02 cells. PA treatment increased the amount of yellow dots. However, PA treatment in combination with celecoxib displayed increased amount of both yellow and red dots indicating autophagic flux was restored partially (Fig. 5c). To further determine the effect of celecoxib on alleviating steatosis, Oil Red O staining and TG kit were used to examine the levels of lipid accumulation in steatosis L02 cells treated with celecoxib. The amount of lipid droplets accumulated in L02 cells and TG content were remarkably decreased in combination of celecoxib and PA treatment groups than those in PA treatment groups (Fig. 5d,e).

### Celecoxib ameliorated NAFLD partially by restoring autophagic flux *in vivo*

**Figure 6:**
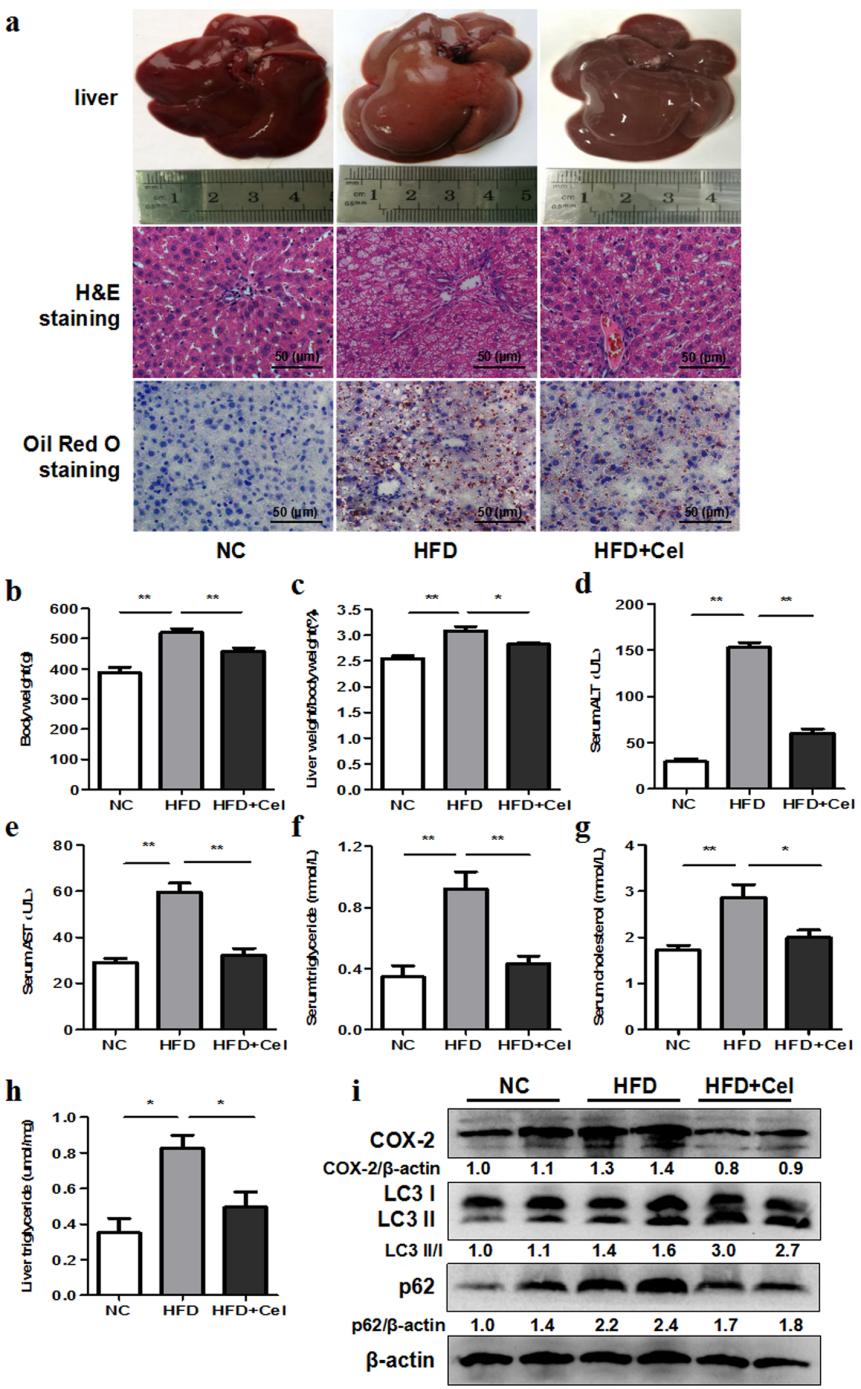
Celecoxib ameliorates NAFLD by restoring autophagic flux *in vivo*. SD rats were fed with normal chow diet (NC, n = 10) or high fat diet (HFD, n = 20) for 8 weeks. At the end of week 4, HFD groups were then randomly intragastricly administrated with celecoxib (HFD + Cel, 20 mg/kg/day, n = 10) or normal saline (n = 10) for another 4 weeks, while the NC groups were intragastricly administrated with normal saline. (**a**) Liver histology was determined by H&E and Oil Red O staining (200X). (**b**–**h**) Body weight, liver weight/body weight, serum levels of ALT, AST, TG, TC, and intrahepatic TG level in HFD + Cel groups were lower than HFD groups (^*^P < 0.05, ^**^P < 0.01). (**i**) HFD induced upregulation of LC3 II/I, p62 and COX-2 levels compared with negative control as indicated by western blot. HFD + Cel groups liver showed higher protein expression of LC3 II/I and lower protein expression of p62 and COX-2 compared with HFD groups. The value under the band is the ratio of the blot and normalized to control.

SD rats were fed with HFD and then treated with celecoxib. As shown in Fig. 6a, the liver HE staining and Oil Red O staining exhibited widespread lipid droplets deposited inside the liver parenchyma cells in HFD rats groups compared with the control groups. However, celecoxib treatment groups exhibited less lipid accumulation. Body weight, liver weight/body weight, serum alanine aminotransferase (ALT), aspartate aminotransferase (AST), total cholesterol (TC), triglyceride (TG), and the liver tissue TG level in celecoxib-treated rats were commonly decreased than control groups (Fig. 6b–h). As presented in Fig. 6i and Supplementary Fig. S9, the expression of COX-2, LC3 II/I and p62 all increased in HFD rats compared with control groups. However, COX-2 and p62 expression was decreased, LC3 II/I expression was increased in celecoxib treatment groups.

## Discussion

NAFLD is a complex multifactorial chronic disease which is charactered by an excessively high accumulation of fat deposits in the liver resulting from causes other than chronic alcohol abuse and drugs^23^. NAFLD is often accompanied by obesity, diabetes, and hyperlipidemia which terribly trouble health. And the pathogenesis of this disease is still unclear. It is generally acknowledged that the theory of “two hits” may provide us a new sight into the process of NAFLD. As is known, NAFLD is closely associated with insulin resistance and lipid metabolism dysfunction, both of which can lead to the accumulation of lipid droplets in hepatocytes (the first hit). Also, lipid oxidation, peroxidation, endoplasmic reticulum stress, inflammation, and endotoxins can further aggravate this disease (the second hit). During the process of the second hit, NAFLD is likely to develop into NASH, cirrhosis, and even cancer. But till today, there is no special medical treatment for NAFLD.

For that reason, it is urgent to find out new ways for NAFLD therapy. In this study, steatosis L02 cells and HFD-fed rats are used as NAFLD models to identify the beneficial effects and underlying mechanisms of celecoxib. We found that celecoxib alleviated lipid accumulation both *in vitro* and *in vivo*. In addition, we found that celecoxib regulated NAFLD partially by restoring autophagic flux.

Autophagy is a protect response to stress resulting from internal and external stimuli in all cells^24,25^. It is generally acknowledged that autophagy plays an important role in maintaining homeostatic state and normal function in cells^26,27^. In the development of NAFLD, autophagy serves as another way for lipid metabolism apart from tricarboxylic acid cycle^18,28^. The process is widely known as lipophagy. A study has demonstrated a relationship between SREBP-2 intracelluar trafficking and impaired autophagic flux in NAFLD. Downregulation of SREBP-2 may result in improved autophagic flux as well as lower accumulation of lipid droplets^20^. Other studies have indicated that inhibition of lipophagy may result in larger number and bigger size of lipid droplets in starved mouse embryonic fibroblasts, while degradation of lipid droplets in mitochondria only requires lipolysis activation^29^. In starved livers, autophagy marker LC3 along with perilipins has been found in lipid droplets and lysosomes^30^. It is likely that a cross-talk between lipolysis and lipophagy plays an important role in the accumulation of lipid droplets in NAFLD. It is also demonstrated that lipid accumulation in hepatocytes blocks autophagic flux. In return, impaired autophagic flux promotes the progress of NAFLD^31^.

We found that autophagic flux was activated on the early stage of steatosis induced by PA in L02 hepatic cells as steatosis maybe a stimuli to cell homeostatic state. Autophagic flux (lipophagy) may act as an attempt to promote lipid turnover. But at the late hour autophagic flux was blocked due to homeostatic imbalance. In addition, rapamycin (autophagy activator) could alleviate lipid accumulation in hepatocytes through activating impaired autophagic flux. Meanwhile, chloroquine (autophagy inhibitor) could aggravate steatosis through blocking autophagic flux. However, it is still unknown how autophagic flux regulates steatosis. Thus it still needs efforts to find out whether autophagic flux regulates lipolysis and lipogenic genes or promotes lipid droplets turnover through autolysosomes.

COX-2, a key enzyme in the process of prostaglandin synthesis, is highly expressed in many diseases, such as cancer, T2DM and NAFLD^32,33^. Celecoxib is a type of non-steroidal anti-inflammatory drug (NSAID) which directly inhibits COX-2. Studies have shown that celecoxib may have functions on cancer, kidney injury as well as NAFLD^34,35^. A study indicated that celecoxib induced and activated autophagy in human prostate cancer PC3 cells in a time and dose-dependent manner. Further study found that autophagy activated by celecoxib played a cytoprotective role in apoptosis^33^. In our study, we also found that COX-2 was overexpressed during steatosis induced by PA in L02 hepatic cells. Both COX-2 siRNA and celecoxib could alleviate lipid accumulation. Then, whether autophagy plays a role in the treatment of celecoxib on alleviating NAFLD is to be answered. We did find out celecoxib could improve NAFLD at least partially by restoring autophagic flux both *in vitro* and *in vivo*. However, it is still unknown how celecoxib regulates autophagic flux. More attention should be paid to find out the underlying mechanisms of celecoxib on regulating autophagy associated gene and the fusion of autophagosome and lysosome.

In conclusion, our present study showed that celecoxib alleviated NAFLD by restoring autophagic flux both *in vitro* and *in vivo*. These findings may help us better understand the mechanisms of NAFLD as well as developing novel therapy strategies.

## Materials and Methods

### Animals

Thirty male Sprague-Dawley (SD) rats were obtained from Beijing Vital River Laboratory Animal Technology (Beijing, China). Six-week-old rats were maintained at 22 ± 2 °C with 60% ± 5% relative humidity under a 12 h light and dark cycle with free access to water and regular chow diet. After one week acclimatization, rats were randomly divided into two groups: control group and HFD (high-fat diet) group. Rats of control group were fed with a normal chow diet (12% kcal fat, 66% kcal carbohydrate, 22% kcal protein) (#1022, from SD rats agent as above) (n = 10). Rats of HFD group were fed with high fat diet (60% kcal fat, 20% kcal carbohydrate, 20% kcal protein) (#D12492, from SD rats agent as above) (n = 20). At the end of week 4, the HFD group rats were then randomly intragastricly administrated with celecoxib (Pfizer, USA) (20 mg/kg/day, n = 10) or normal saline (n = 10) for another 4 weeks along with HFD, while the control group rats were intragastricly administrated with normal saline. At the end of week 8, rats were sacrificed for 12 h fasting. Blood samples were collected and livers were removed and weighed for further study. Animal experiments were approved by Ethics Committee of Tongji Hospital and all experiments were performed in accordance with relevant guidelines and regulations for care and use of laboratory animals.

### Biochemical analyses

Blood biochemical parameters, including serum ALT, AST, TC, and TG were measured by kits (#C009-2, #C010-2, #A111-1, and #A110-1, Jiancheng Bioengineering Institute, Nanjing, China). Liver TG was determined by kit (#E1013, Applygen Technologies, Beijing, China).

### Cell line, culture, and treatment

The human hepatic cell line L02 was maintained at Institute of liver diseases, Tongji Hospital, Wuhan, China. The L02 cells were cultured in RPMI-1640 medium containing 10% fetal calf serum (#10100147, Invitrogen Gibco, Carlsbad, CA, USA) and incubated in a 5% CO_2_ incubator at 37 °C. Celecoxib, rapamycin, and chloroquine (HY-14398, HY-17589, and HY-10219, respectively, MedChemExpress, Monmouth Junction, NJ, USA) were added into the culture at indicated concentrations and timepoints.

### Preparation of palmitate

0.2 mmol sodium palmitate (PA) (#FIZ6I-PJ, Tokyo Chemical Industry, Japan) was mixed in 0.1 M 2 ml NaOH at 70 °C. 5% bovine serum albumin (BSA) was prepared in 38 ml phosphate-buffered saline (PBS). The solution of PA was conjugated to 5% BSA in a 50 °C water bath for 2 h. The stock concentration of PA is 5 mM. Finally, the PA solution filtered and stored at −20 °C.

### Oil Red O staining

The lipid accumulation in cells or liver is detected by Oil Red O staining kit (#D027, Jiancheng Bioengineering Institute, Nanjing, China) according to the manufacturer’s instructions.

### Cell viability assay

Cells (5 × 10^3^ cells/well) were seeded in 96-wells plate. After treatment, cells were incubated with a mixed content (10 μl CCK8 reagent +90 μl RPMI-1640 medium) (#P5090, Promoter, Wuhan, China) at 37 °C for 2 h. Finally, the value was measured at 450 nm OD.

### Transient transfection

L02 cells were transfected with RFP-GFP-LC3 double fluorescence lentivirus (#GPL2001, Genechem, Shanghai, China) at 20–30% confluence according to the manufacturer’s protocol. We got the stable expression transfectant by adding puromycin. Then the L02 cells were treated with PA or celecoxib. To knockdown COX-2, siRNAs were transfected into cells using Lipofectamine2000 (#11668-019, Invitrogen, Carlsbad, CA, USA) according to the manufacturer’s protocol. The siRNA sequences (#1299001, Invitrogen, Carlsbad, CA, USA) used as follow: si-1 5′-GAAGCCUUCUCUAACCUCUCCUAUU-3′ and si-2 5′-AUCAGUAUUAGCCUGCUUGUCUGGA-3′.

### Quantitative real-time PCR

Total RNA was extracted by TRIzol reagent (#15596018, Invitrogen, Carlsbad, CA, USA). Reverse-transcribed complementary DNA was synthesized using the PrimeScript RT reagent kit (#RR036A, TaKaRa, Otsu, Japan). Real-time PCR was performed using SYBR Premix ExTaq (#RR820A, TaKaRa, Otsu, Japan) on an ABI StepOne Real-Time PCR System (Applied Biosystem, Carlsbad, CA, USA). Difference between samples was determined by the 2^−ΔΔCt^ method. Primers were designed using NCBI Primer-BLAST. COX-2, Forward Primer 5′-CTGGCGCTCAGCCATACAG-3′ and Reverse Primer 5′-CGCACTTATACTGGTCAAATCCC-3′.

### Western blot

Total protein from cells and liver were extracted, concentration was quantitated using BCA protein quantification kit (#P5026, Promoter, Wuhan, China). Protein in PVDF membranes were incubated with primary antibody at 4 °C overnight, LC3 I/II and p62 (#4108, #5114, both 1:1000, Cell Signaling Technology, Boston, USA), COX-2 and β-actin (#P1023-01, #P0015, 1:1000 and 1:4000, Promoter, Wuhan, China) followed by anti-rabbit IgG (#P0009, 1:4000, Promoter, Wuhan, China) for 1 h at room temperature. Immunoreactive bands were visualized with ECL kit (#P5876, Promoter, Wuhan, China) and pictured by GeneGnome5 Chemiluminescence Series Image Capture (Syngene, Frederick, MD, USA) according to manufacturers’ instructions. All the western blot experiments were repeated at least two times. Blot densitometric analysis was done by ImageJ software (NIH).

### Statistical analysis

Results were presented as mean ± SD. For comparisons between two groups, Student’s t-tests were used. For multiple groups, ANOVA analysis with or without repeated measures was used. *P* < 0.05 was considered significant.

## Electronic supplementary material

Supplementary information
