# Supplementary material for: Celecoxib alleviates nonalcoholic fatty liver disease by restoring autophagic flux
Source: Sci Rep. 2018 Mar 7;8:4108. doi: 10.1038/s41598-018-22339-0 (PMC5841322; doi:10.1038/s41598-018-22339-0)
Supplement: Supplementary file 1 — Supplementary information [file 41598_2018_22339_MOESM1_ESM.pdf]

Celecoxib alleviates nonalcoholic fatty liver disease by restoring autophagic flux

Cong Liu[1], Lian Liu[1], Hai-Dan Zhu[1], Jia-Qi Sheng[1], Xiao-Li Wu[1], Xing-Xing He[1], De-An Tian[1], Jia-Zhi Liao[1]*, Pei-Yuan Li[1]*

[1]Division of Gastroenterology, Tongji Hospital, Tongji Medical College, Huazhong University of Science and Technology, Wuhan 430030, China.

*Correspondence and requests for materials should be addressed to J.-Z.L. (email: liaojiazhi@sina.com) or P.-Y.L. (email: pyli@tjh.tjmu.edu.cn)

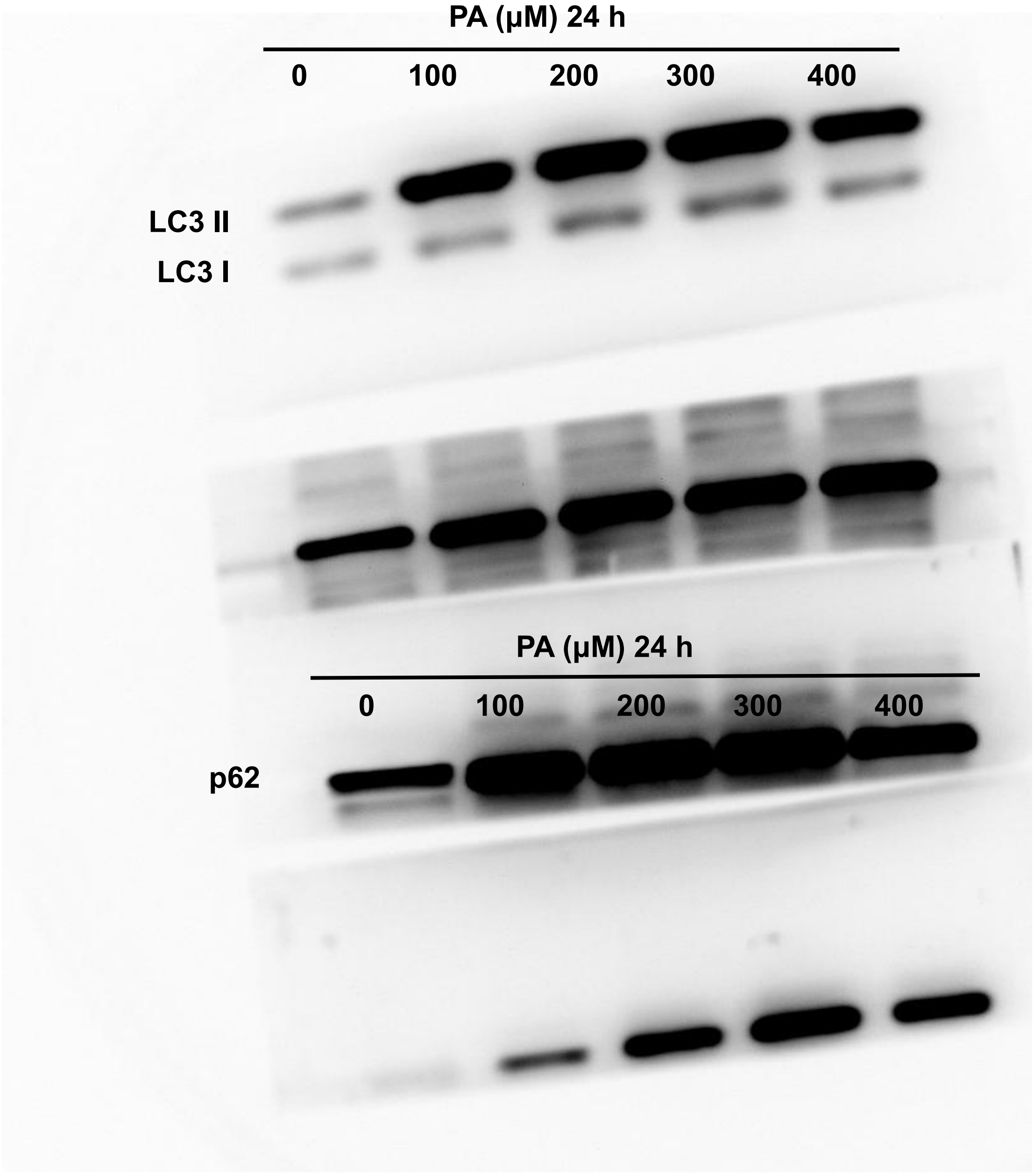

Figure 1. Effects of PA on autophagic flux. L02 cells exposed to different concentrations (100-400 μM) of PA for 24 h. PA induced upregulation of LC3 II/I and p62 protein levels as indicated by western blot.

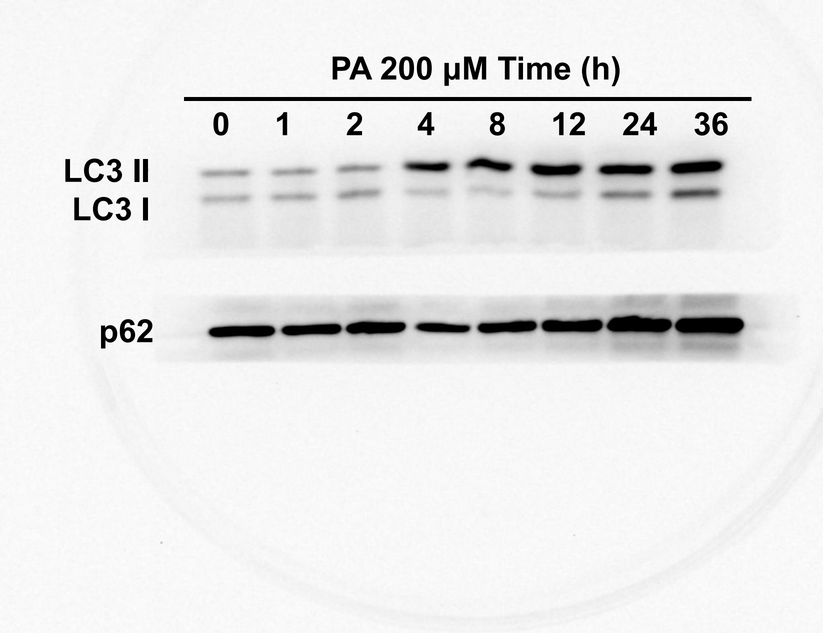

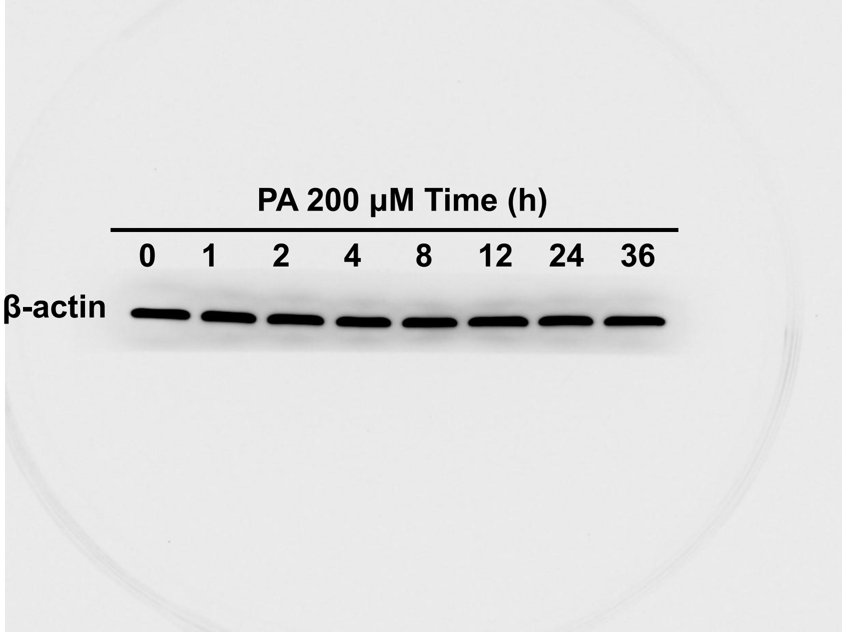

Figure 2. Effects of PA on autophagic flux. L02 cells were treated with PA (200 μM) for different time (0-36 h). PA induced upregulation of LC3 II/I and downregulation of p62 protein levels at 4 h, 8 h while upregulation of LC3 II/I and p62 protein levels at 24 h, 36 h indicating that autophagic flux was activated on the early stage but blocked at the late hour.

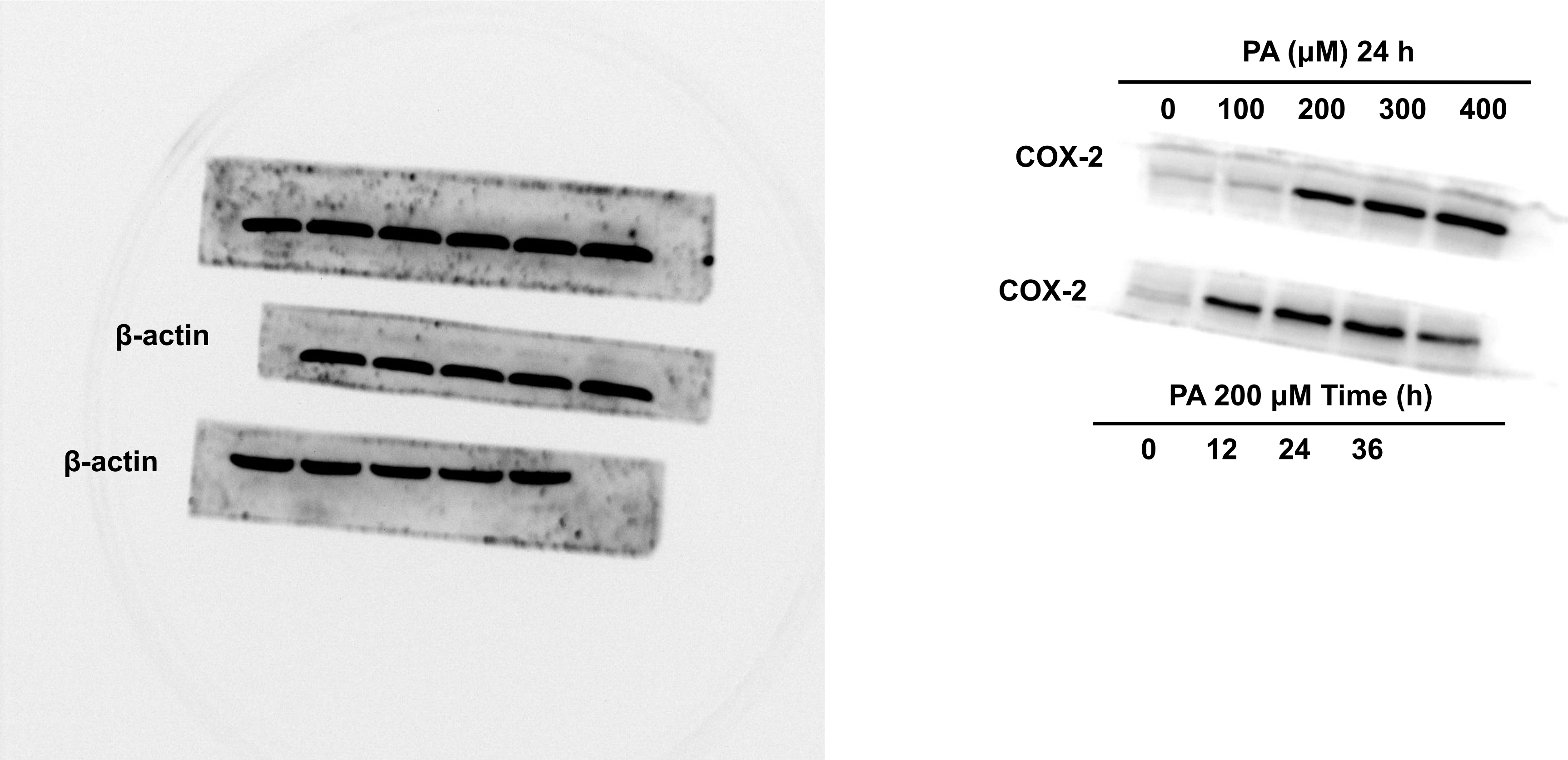

Figure 3. Effects of PA on COX-2. L02 cells were treated with PA of different concentrations (100-400 μM) for different time (12-36 h). PA induced upregulation of COX-2 protein levels.

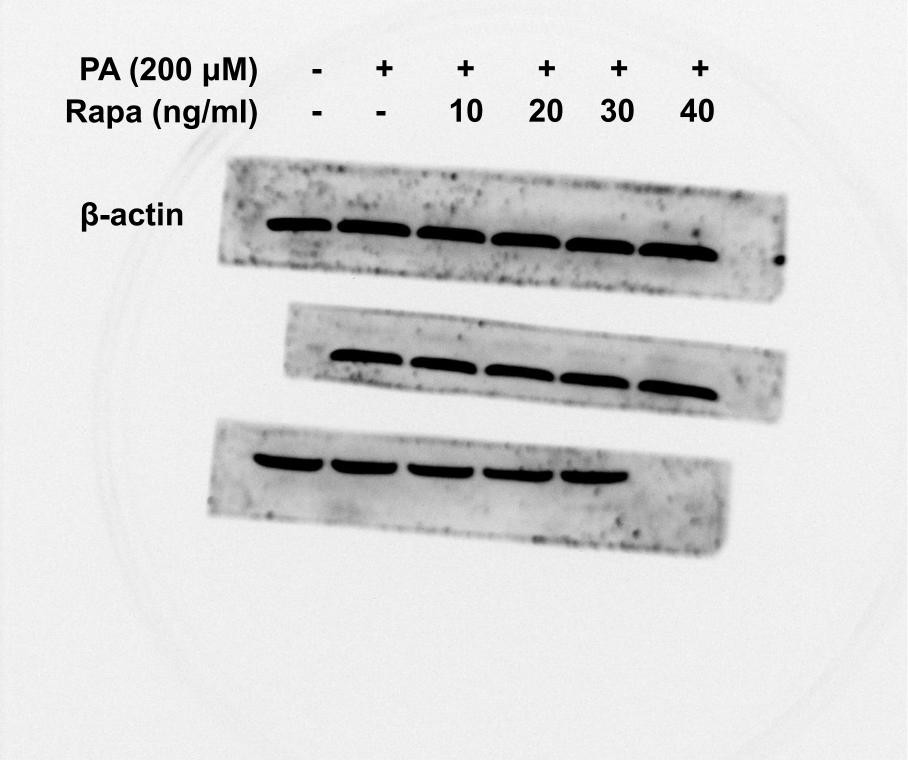

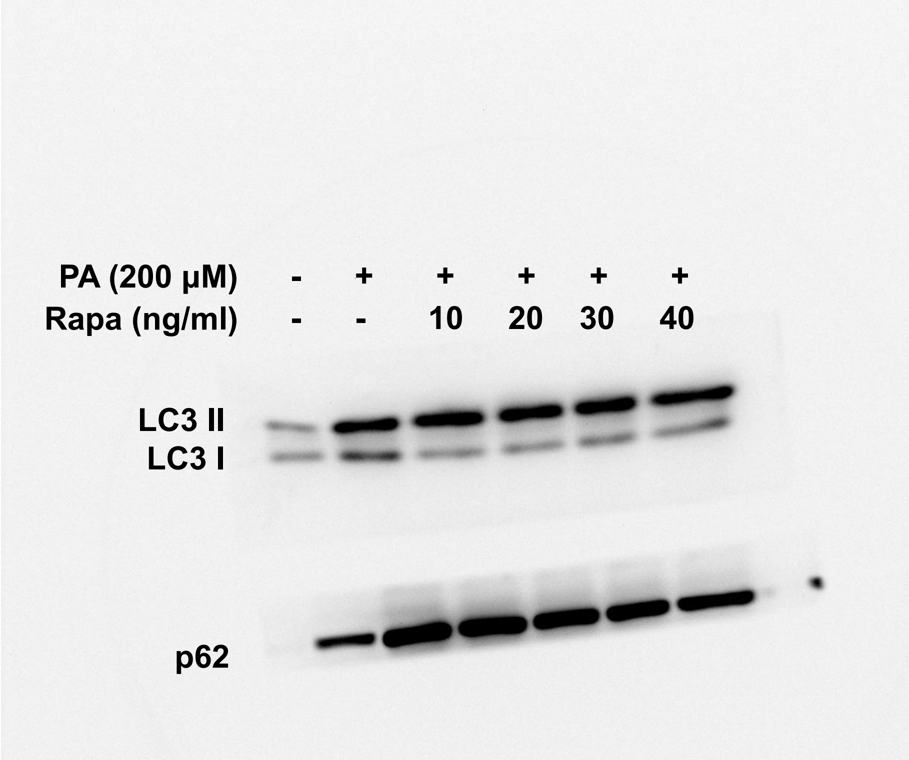

Figure 4. Autophagy plays an important role in steatosis. L02 cells exposed to PA (200 μM) with different concentrations of rapamycin (Rapa) for 24 h. PA induced higher protein expression of LC3 II/I and p62 compared with control as indicated by western blot. Rapamycin combined with PA treatment induced higher LC3 II/I protein levels and lower p62 protein levels compared with PA treatment.

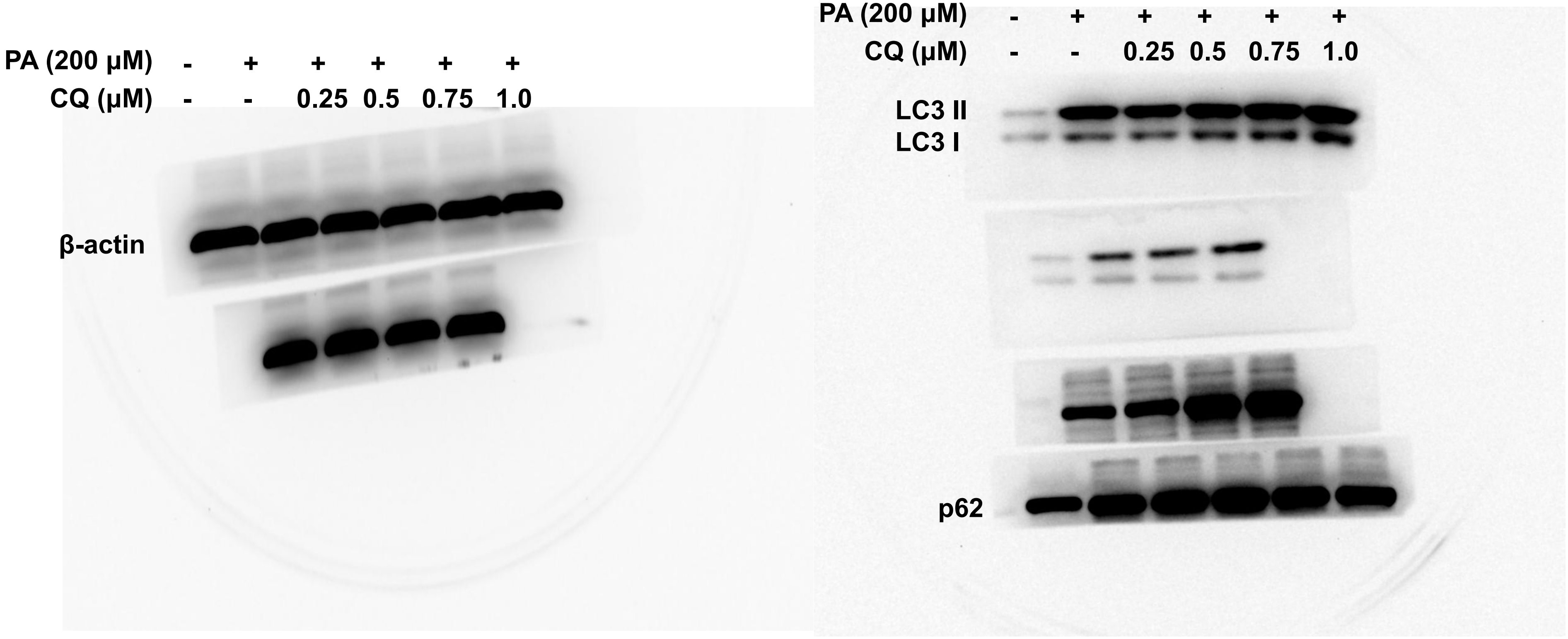

Figure 5. Autophagy plays an important role in steatosis. L02 cells exposed to PA (200 μM) with different concentrations of chloroquine (CQ) for 24 h. PA induced higher protein expression of LC3 II/I and p62 compared with control as indicated by western blot. Chloroquine combined with PA treatment induced higher p62 protein levels compared with PA treatment.

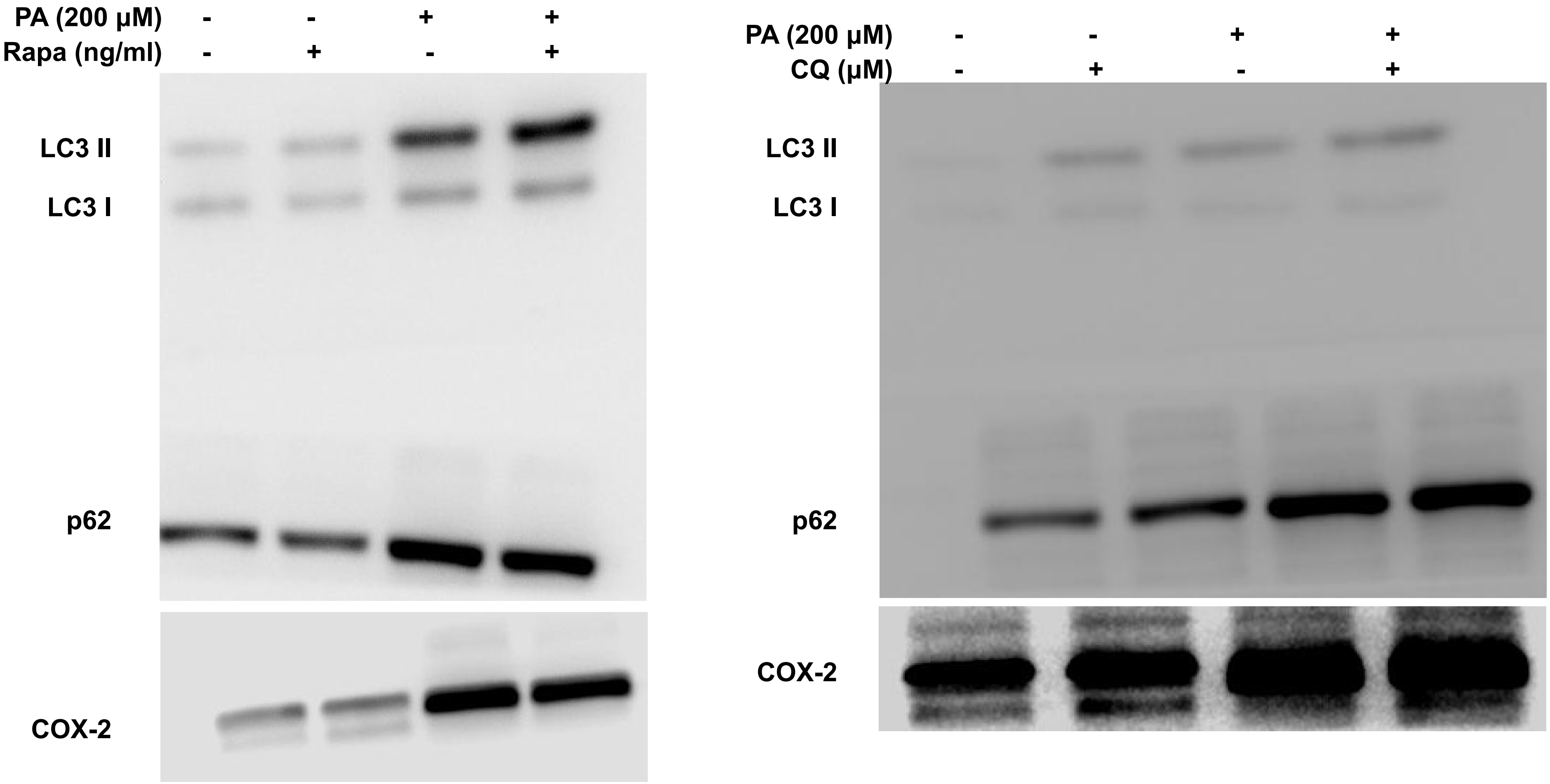

Figure 6. Regulation of autophagy flux has no obvious influence on COX-2 in turn. L02 cells exposed to PA (200 μM) with rapamycin (20 ng/ml) or chloroquine (0.5 μM) for 24 h. PA induced remarkable increase of COX-2. Both rapamycin and chloroquine could regulate autophagy flux, but none of them had obvious influence on the COX-2 protein levels.

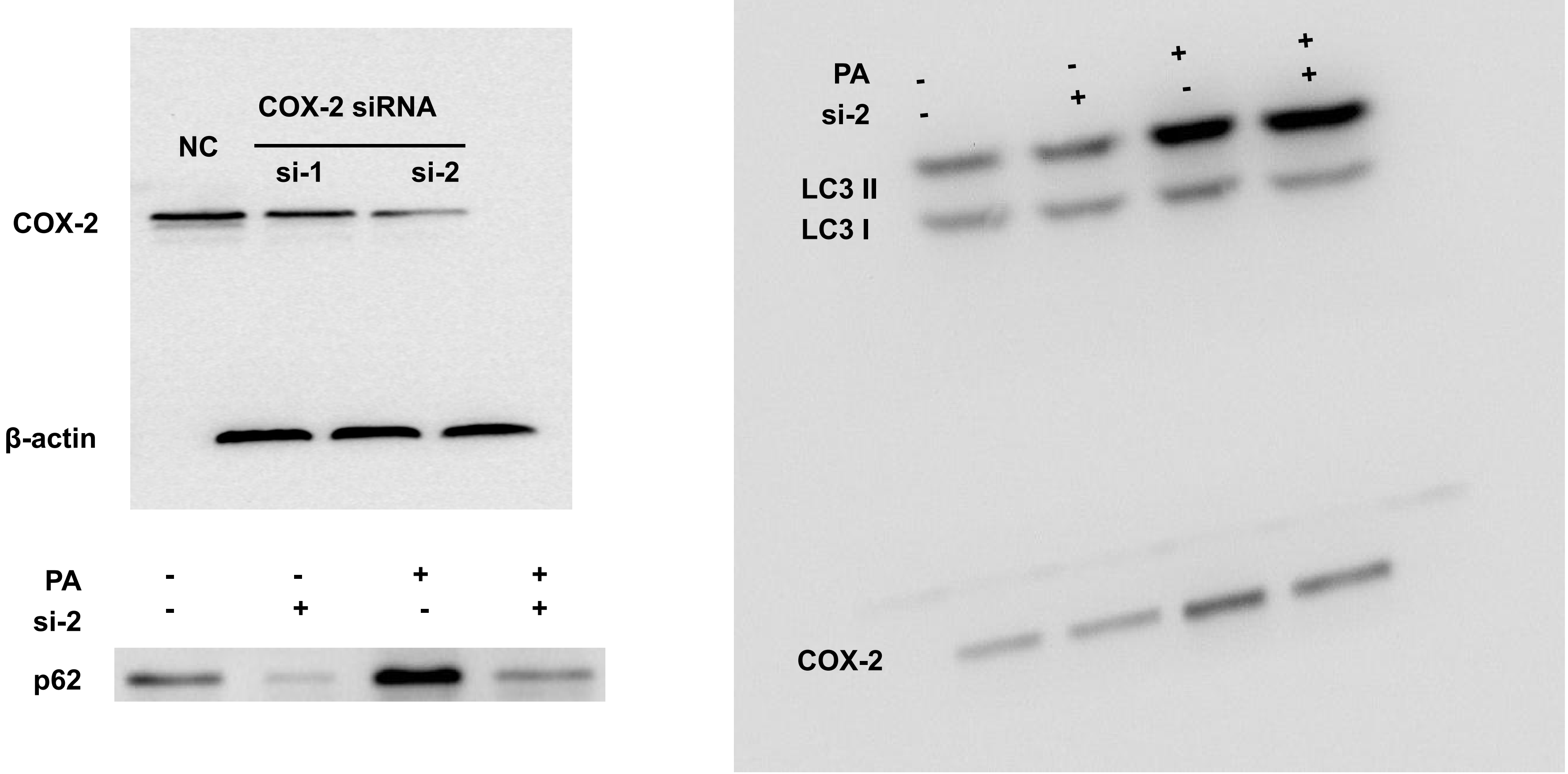

Figure 7. Downregulation of COX-2 induces autophagic flux *in vitro*. COX-2 was knocked down with COX-2 siRNA transfection (si-1 or si-2) in L02 cells, then western blot were performed for confirmation. After treated with PA (200 μM) for 24 h, western blot of COX-2 siRNA transfected cells showed higher protein expression of LC3 II/I and lower protein expression of p62 than negative control (NC).

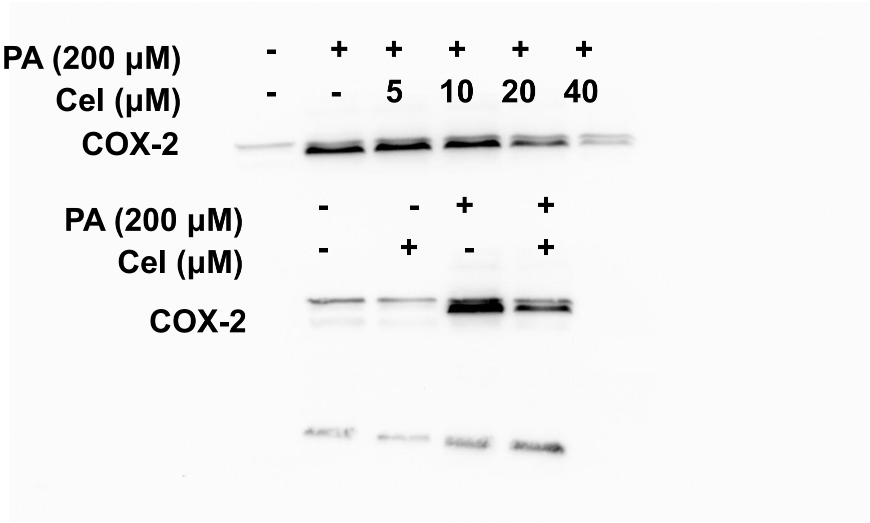

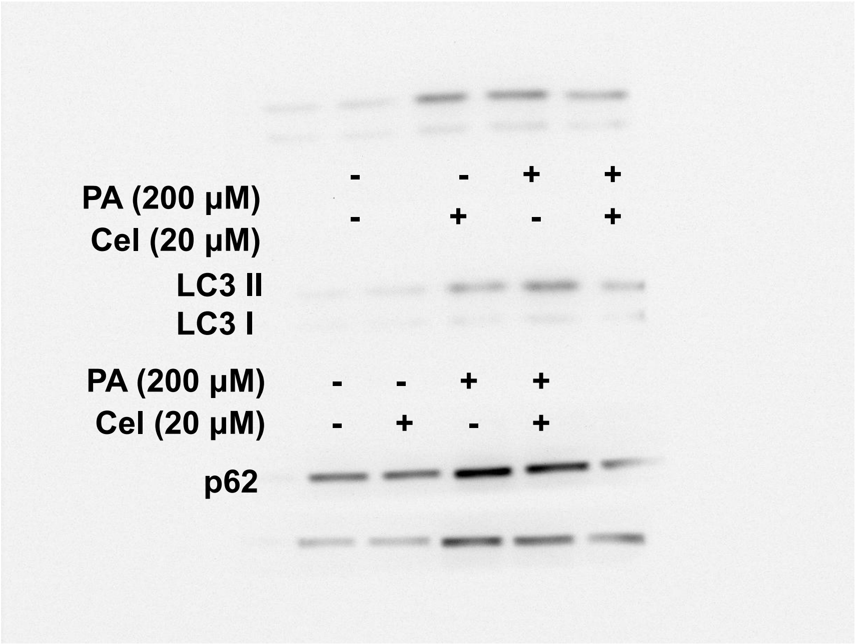

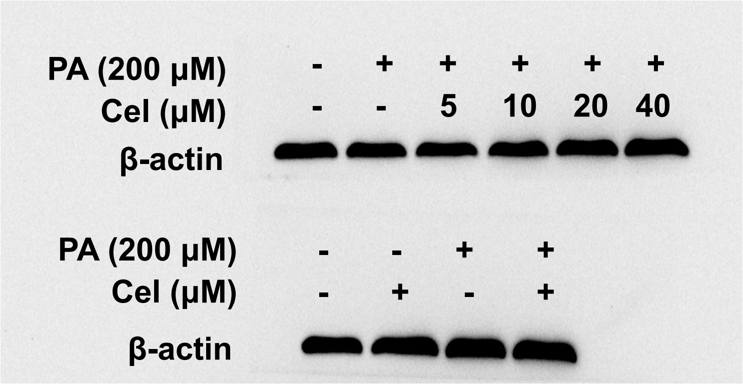

Figure 8. Celecoxib alleviates steatosis by restoring autophagic flux *in vitro*. L02 cells exposed to PA (200 μM) with different concentrations of celecoxib (Cel, 5-40 μM) for 24 h. Celecoxib decreased protein expression of COX-2 compared with control as indicated by western blot. L02 cells were treated with PA (200 μM) and celecoxib (20 μM) for 24 h. Celecoxib combined with PA treatment induced higher protein expression of LC3 II/I and lower protein expression of p62 compared to PA treatment.

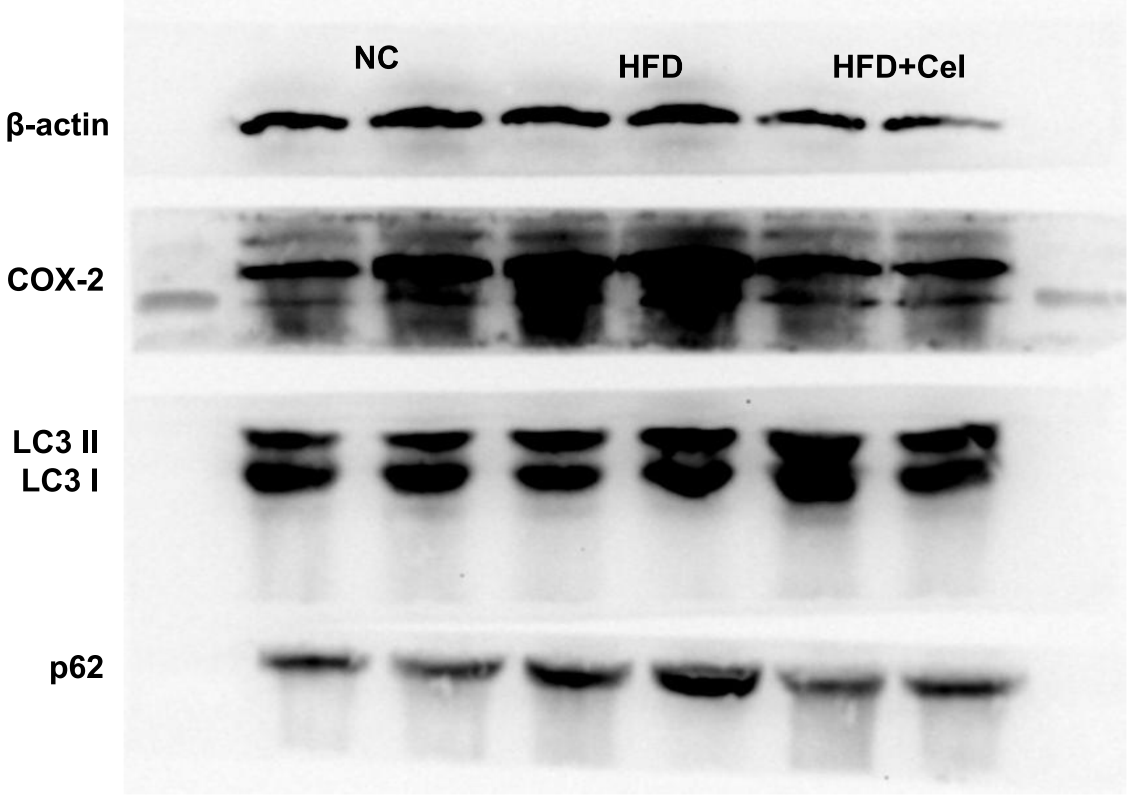

Figure 9. Celecoxib ameliorates NAFLD by restoring autophagic flux *in vivo*. SD rats were fed with normal chow diet (NC, n=10) or high fat diet (HFD, n=20) for 8 weeks. At the end of week 4, HFD groups were then randomly intragastricly administrated with celecoxib (HFD+Cel, 20 mg/kg/day, n=10) or normal saline (n=10) for another 4 weeks, while the NC groups were intragastricly administrated with normal saline. HFD induced upregulation of LC3 II/I, p62 and COX-2 levels compared with negative control as indicated by western blot. HFD+Cel groups liver showed higher protein expression of LC3 II/I and lower protein expression of p62 and COX-2 compared with HFD groups.
